# Data-Driven Remaining Useful Life Prediction for Pt–Rh Thermocouples Using an Extended Kalman Filter

**DOI:** 10.3390/s26051483

**Published:** 2026-02-26

**Authors:** Na Li, Siyang Dai, Yi Liu, Yunlong Zhu, Jitao Li, Xiaojin Huang

**Affiliations:** 1 State Key Laboratory of Chemical Safety, College of Mechanical and Electrical Engineering, China University of Petroleum (East China), Qingdao 266580, China; 2 State Key Laboratory of Chemical Safety, SINOPEC Research Institute of Safety Engineering Co., Ltd., Qingdao 266000, China; 3 State Key Laboratory of Chemical Safety, College of Chemistry and Chemical Engineering, China University of Petroleum (East China), Qingdao 266580, China; 4 Institute of Nuclear and New Energy Technology, Key Laboratory of Advanced Reactor Engineering and Safety of Ministry of Education, Tsinghua University, Beijing 100084, China; lijt22@mails.tsinghua.edu.cn (J.L.);

**Keywords:** back-propagation neural network, extended Kalman filter, Pt–Rh thermocouple, remaining useful life

## Abstract

Platinum (Pt)–Rhodium (Rh) thermocouples are widely used in industrial processes such as chemical and nuclear power production, serving as one of the most common temperature measuring instruments and playing a vital role in real-time condition monitoring. However, the measurement accuracy can be affected by harsh high-temperature operating environments, which may cause measurement drift or even functional failure. To address this challenge, and considering the very slow drift of Pt–Rh thermocouples over long time scales, a back-propagation neural network (BPNN) is introduced to compensate for the nonlinear error introduced by the linearization step of the extended Kalman filter (EKF). This combined algorithm enhances the accuracy of remaining useful life (RUL) prediction for Pt–Rh thermocouples. First, based on the Seebeck effect and vapor-transport theory, a degradation model for Pt–Rh thermocouples operating at high temperatures was developed. The simulation results of the degradation model align with laboratory degradation test data, confirming the validity of the model. Subsequently, the improved RUL prediction algorithm was compared with other methods. The results show that the EKF–BPNN hybrid approach provides better prediction accuracy for objects with slow degradation and weak nonlinearity, with MAE 0.0016%, RMSE 0.0019%, MAPE 0.039%, $R^{2}$ 0.9833, respectively. Algorithms with strong nonlinear estimation capability introduce larger errors and are not suited for RUL prediction of Pt–Rh thermocouples. Therefore, the proposed hybrid EKF–BPNN algorithm is optimal for RUL prediction of Pt–Rh thermocouples degrading under high temperature conditions.

## 1. Introduction

Thermocouples are widely used for temperature measurement in industrial settings and are the most prevalent type of temperature sensor. They offer several advantages, including low cost, a broad measurement range, robust and stable construction, and fast response times. In a 10 MW high-temperature gas-cooled reactor, thermocouples are installed at both the outlet and inlet of the steam generator to monitor coolant temperature. Additionally, 40 measurement points are distributed within the reactor core components to track temperature profiles of graphite and metallic elements, while 57 thermocouples are mounted on the pressure vessel surface to observe temperature variations [1]. During thermal fatigue testing of aircraft components, thermocouples provide real-time and accurate temperature data [2]. Accurately measuring the helium gas temperature at the outlet of the fuel region of a very high-temperature gas-cooled reactor using thermocouples is critical. This ensures proper fuel performance under high-temperature conditions, maintains the integrity of materials within strict operational limits, and supports the safe operation of the reactor [3]. In industrial processes, thermocouple-based instruments serve as a direct monitoring tool for operators to assess equipment status and process normality. In essence, a thermocouple instrument functions like the “eyes” of the operating personnel. Loss of thermocouple measurement capability would prevent real-time awareness of equipment operating conditions, potentially leading to serious production incidents.

The working principle of a thermocouple is based on the Seebeck effect. When a temperature difference exists between the hot and cold ends, an internal electric field is generated along the alloy wires in the direction of decreasing temperature gradient. The voltage produced between the two wires is then converted to determine the temperature at the hot end. At a more microscopic level, electrons at the hot end possess higher energy than those at the cold end, leading to net diffusion of electrons from the hot end toward the cold end within the conductor. As electrons move to the cold end, positively charged metal ions remain at the hot end. When sufficient electrons accumulate at the cold end, an internal electric field is established that opposes further electron diffusion. An equilibrium is eventually reached internally. The resulting thermoelectric voltage per unit temperature difference is defined by the Seebeck coefficient [4]. However, under harsh operating conditions, the Seebeck coefficient of thermocouples can be adversely affected, leading to measurement inaccuracies or complete functional failure. Therefore, predicting the degradation of thermocouples under severe operational conditions has become an urgent priority. Research findings in this area will play a crucial and positive role in maintaining the normal and safe operation of industrial processes.

The prediction of RUL has long been a focus of industrial research, with extensive studies conducted on RUL estimation methods for various systems and equipment. Key objects of such research include gears [5,6], bearings [7,8], aeroengines [9,10], lithium batteries [11,12], and circuits [13,14]. A thermocouple instrument consists of a primary sensor and a secondary instrument. The primary sensor refers to the thermocouple sensing element. The part where the thermoelectric effect generates the electromotive force (Emf). This component operates directly in harsh environments and is most susceptible to degradation. Since it is in direct contact with the measured object, it serves as the most immediate means of monitoring equipment operational status. Its measurement accuracy is crucial for the safety of industrial operations. The secondary instrument typically comprises analog and digital circuits that perform functions such as filtering, amplification, and analog-to-digital conversion. Its role is to convert the non-standard signal generated by the primary sensor into a standardized electrical signal for transmission to a computing server. Usually operating under normal environmental conditions away from the equipment, the secondary instrument rarely experiences performance degradation. Therefore, performance degradation in a thermocouple instrument is primarily attributable to the deterioration of the primary sensor.

Model-based methods are suitable for scenarios where a reasonably accurate degradation model of the physical object can be constructed. Currently, among the widely applied model-based methods in RUL prediction are Kalman filter (KF) [15,16] and particle filter (PF) [17,18] methods. KF is straightforward to implement but requires an accurate model and precise initial states as prerequisites. Its capability to estimate states in nonlinear models is relatively limited. To address this, long short-term memory (LSTM) networks have been integrated with EKF to enhance nonlinear estimation performance. Further improvements, such as incorporating a squared gain to boost noise robustness, have led to superior prediction of the state of charge (SOC) for lithium-ion batteries [19]. Another enhancement involves upgrading the first-order Taylor expansion used in EKF to a second-order Taylor expansion within an adaptive EKF framework, reducing truncation error and improving SOC estimation accuracy [20]. Additionally, combining model parameter identification with EKF has also been proposed to enhance SOC estimation precision in nonlinear systems [21]. PF, a Monte Carlo-based approximate Bayesian filtering algorithm, demonstrates better performance than KF in handling nonlinear estimation problems, though at the cost of higher computational resource consumption. The grey model has been used to complement the degradation model of lithium-ion batteries, improving the RUL prediction performance of PF [22]. Furthermore, the particle swarm optimization algorithm has been applied to obtain more accurate posterior estimates in PF, helping to avoid local optima and thereby enhancing RUL prediction accuracy [23].

In recent years, with the advancement of artificial neural networks, research has increasingly focused on data-driven neural network prediction methods. Representative algorithms include BPNN [24,25], gated recurrent unit (GRU) network [26,27], and LSTM network [28,29]. Based on simulated fatigue crack-propagation data, a method combining a genetic algorithm with a BPNN has been used to predict stress intensity factors in pipelines. By integrating these stress intensity factors with physical laws, the RUL of pipelines can be characterized [30]. To address limitations in data-adaptive weighting and prediction accuracy in models combining convolutional neural networks (CNNs) and GRU, a double convolutional attention mechanism was incorporated into a CNN-GRU hybrid prediction framework. This mechanism dynamically allocates weights to features, and its effectiveness in capturing degradation information and improving prediction accuracy was validated using aircraft engine datasets [31]. Additionally, a hybrid LSTM-CNN network that integrates both statistical and physical features has been proposed to predict RUL of lithium-ion batteries. This approach demonstrates significantly improved prediction performance compared to traditional LSTM-based models [32].

In industrial applications, thermocouples are the most widely used temperature instruments, yet their measurement degradation tends to be slow, with insignificant drift and limited characteristic data. Nonlinear methods capable of predicting RUL of components such as engines, bearings, gears, and lithium-ion batteries, which exhibit strongly nonlinear degradation behavior, may not be directly applicable to thermocouple RUL prediction. Furthermore, due to the scarcity of degradation data for thermocouples, purely data-driven approaches often struggle to train reliable degradation models. Therefore, in this paper, we investigate an RUL prediction algorithm that combines EKF with BPNN, tailored to the degradation characteristics of thermocouple instruments. This hybrid approach is designed to better address RUL prediction for objects with weak nonlinear features and long-term degradation processes. For algorithms such as the UKF, EPF, and PF, particle sampling during the prediction process can introduce additional errors. In contrast, purely data-driven RUL methods lack the interpretability and deterministic prediction capability offered by model-based approaches. Furthermore, by compensating for the error terms of the EKF, the BPNN refines EKF-based RUL predictions, thereby further enhancing overall prediction accuracy. The major contributions of this paper are fourfold:

(1) To address the degradation characteristics of thermocouple instruments, an EKF–BPNN RUL prediction algorithm is proposed, which builds upon the model-based EKF method and employs a BPNN for bias compensation. The model-based component provides interpretability, while the data-driven compensation helps correct errors introduced during the linearization of the nonlinear model, thereby improving prediction accuracy.
(2) A physical degradation model for type B Pt–Rh thermocouples was developed based on the Seebeck effect and vapor transport theory. The numerical simulation results from this degradation model align with experimental degradation data obtained from laboratory tests on type B Pt–Rh thermocouples aged at 1324 °C for 500 h, thereby validating the model. The simulated degradation curve is subsequently used as a reference for RUL prediction.
(3) The performance of the proposed EKF–BPNN prediction algorithm was evaluated and compared with nonlinear estimation methods such as EKF, PF, unscented Kalman filter (UKF), and extended Kalman particle filter (EPF) using metrics including mean absolute error (MAE), root mean square error (RMSE), mean absolute percentage error (MAPE), and the coefficient of determination ($R^{2}$). The results confirm that the proposed method achieves higher accuracy in RUL prediction compared to existing approaches.
(4) Finally, the findings demonstrate that for objects with weak nonlinearity and slow degradation, such as thermocouple instruments, the proposed EKF–BPNN algorithm is not only suitable but also effectively compensates for the limitations of purely model-based methods, leading to better RUL prediction performance. Additionally, it is shown that RUL prediction algorithms with strong nonlinear modeling capabilities may introduce greater errors when applied to objects with weak nonlinear features, thereby worsening prediction outcomes. Hence, tailored solutions should be applied when addressing degradation problems across different types of objects.

The remainder of this paper is structured as follows: Section 2 introduces the degradation mechanism and the numerical modeling process for Pt–Rh thermocouples under high-temperature conditions. Section 3 describes the proposed RUL prediction method that combines EKF with BPNN. Section 4 presents the experimental analysis, including validation results for the degradation model of type B Pt–Rh thermocouples and a performance comparison between the EKF–BPNN approach and other nonlinear RUL prediction methods for estimating the RUL of Pt–Rh thermocouple instruments. Section 5 concludes the paper.

## 2. Pt–Rh Thermocouple Degradation Model

### 2.1. Vapor Transport

**Figure 1:**
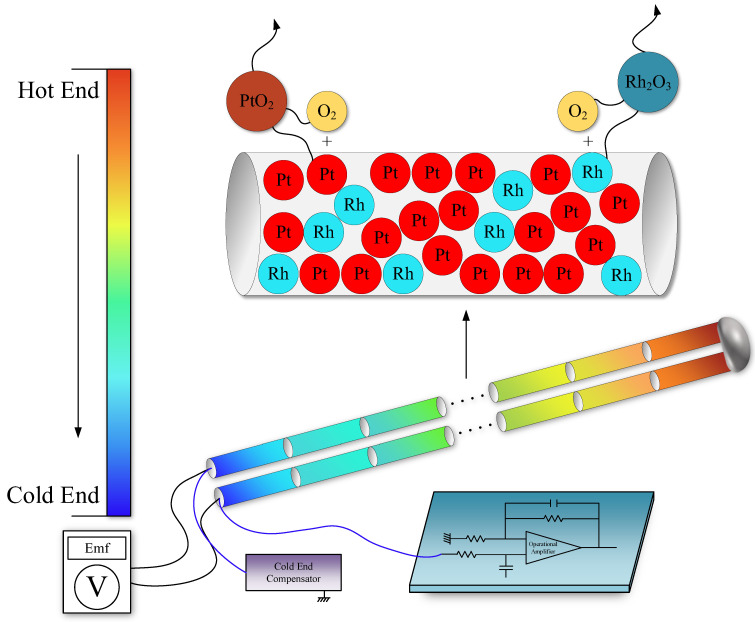
Thermocouple instrument structure and steam transport degradation diagram.

Thermocouples measure temperature using the thermoelectric effect, discovered by German physicist Thomas Johann Seebeck in 1821. When a temperature difference exists between the two ends of a metal wire, an internal electric field is generated along the wire. A thermocouple is therefore composed of two wires made of different alloys. The joined end, known as the hot junction, is placed at the measurement point, while the open ends—the cold junction—output the voltage produced by the thermoelectric effect. This voltage signal is first passed through a low-pass filter to remove noise, then amplified to obtain a standardized electrical signal suitable for industrial applications. The output signal of a thermocouple is a voltage that corresponds directly to temperature, with standard reference tables typically calibrated at a cold-end temperature of 0 °C. In practice, however, the cold end is influenced by ambient temperature and rarely remains at 0 °C. Therefore, cold-end compensation is applied to correct the voltage accordingly. As illustrated in Figure 1, two dissimilar alloy wires are joined at the hot end, while their cold ends are respectively connected to a cold end compensation and a filtering amplification circuit. Due to thermocouple degradation at high temperatures, the two alloy wires degrade differently with temperature, altering their material properties. This leads to drift in both the output Emf and the measured temperature over time.

Compared to base-metal thermocouples, noble-metal thermocouples offer advantages such as chemical inertness, high electrical conductivity, corrosion resistance, longer service life, higher accuracy, and better temperature measurement consistency. Degradation in thermocouples manifests as drift in the output voltage, which results from changes in the thermoelectric homogeneity of the two alloy wires. In Pt–Rh thermocouples with different Rh mass fractions, degradation at high temperatures essentially involves a change in the alloy composition—specifically, the Rh mass fraction in each wire. This alters the thermoelectric properties of the alloys, i.e., their Seebeck coefficients, leading to drift in the output Emf [33]. At high temperature operating conditions, changes in the Rh mass fraction are related to oxidation reactions and the transport of oxide vapors. When thermocouples are used in air at high temperatures, metal atoms on the alloy surface react with oxygen to form oxides. These gaseous metal oxides are then transported via oxide vapor transport [34], as illustrated in Figure 1. The solid Pt and Rh on the alloy surface oxidize in the presence of oxygen. Due to the high temperature, the resulting oxides evaporate into the gaseous phase and detach from the wire surface. Notably, oxide vapor transport occurs primarily along the radial direction of the wire and is confined to the surface layer, with negligible axial transport along the wire length [35]. Given that Pt and Rh atoms are uniformly distributed within the alloy, the Rh mass fraction at the surface is the same as in the bulk. The vapor transport process can be summarized as follows:

① $O_{2}$ molecules diffuse toward the alloy surface;
② $O_{2}$ dissociates and adsorbs on the surface, reacting with metal atoms to form oxide molecules;
③ At high temperature, the oxide molecules evaporate and desorb from the surface;
④ The oxide molecules are transported away from the surface, thereby altering the Rh mass fraction and degrading the thermoelectric homogeneity of the wire.

In high temperature environments, pure Pt is highly susceptible to contamination by other metallic elements. The introduction of foreign metals into the Pt wire due to high-temperature doping can significantly degrade thermocouple performance. However, if a certain amount of Rh is already present in the Pt, the impact of contamination-induced compositional changes is considerably smaller than that observed in pure Pt [36]. Among noble-metal thermocouples such as types B, R, and S, the negative leg of type R and type S thermocouples consists of pure Pt. Consequently, under high temperature conditions, type B thermocouples exhibit superior performance compared to types R and S and are better suited for use in such demanding environments. Therefore, this study focuses primarily on type B Pt–Rh thermocouples.

In high-temperature, oxygen-containing environments, Pt-Rh thermocouple alloy wires undergo oxidation reactions. The subsequent evaporation of metal oxides from the wire surface alters the alloy composition, leading to a change in the Seebeck coefficient [37]. However, the onset evaporation temperatures and evaporation rates for Pt oxides and Rh oxides differ. Therefore, the analysis accounts for the distinct evaporation characteristics of each oxide. Moreover, in Pt–Rh thermocouples, Pt is more prone to vapor transport than Rh [38]. This is because the less volatile Rh sesquioxide ($Rh_{2}O_{3}$) forms a surface layer over Rh dioxide ($RhO_{2}$), thereby inhibiting the vapor transport of Rh oxides [39].

The oxidation process of Pt proceeds as follows [40]:

(1) $$Pt\left(\mathrm{solid}\right)+O_{2}\left(\mathrm{gas}\right)\to PtO_{2}\left(\mathrm{gas}\right),$$

The oxidation of Rh proceeds through the formation of two compounds, as described below [33]:

(2) $$Rh\left(\mathrm{solid}\right)+O_{2}\left(\mathrm{gas}\right)\to RhO_{2}\left(\mathrm{gas}\right),$$

(3) $$4Rh\left(\mathrm{solid}\right)+3O_{2}\left(\mathrm{gas}\right)\to 2Rh_{2}O_{3}\left(\mathrm{gas}\right),$$

Pt-group metal solid oxides are unstable above a critical temperature under standard atmospheric pressure and undergo dissociation, i.e., a vapor transport process [34]. In an oxygen-containing atmosphere, the evaporation of $PtO_{2}$ begins at approximately 650 °C. Below this temperature, $PtO_{2}$ exhibits negligible mass loss [41,42]. In contrast, $Rh_{2}O_{3}$ starts to evaporate around 1140 °C, significantly higher than the evaporation temperature of Pt oxides. Consequently, Rh is less prone to evaporation, and in Pt–Rh alloy wires, Pt evaporates preferentially over Rh [43].

The vapor transport rate is defined as [34]:

(4) $$v=-\frac{1}{S}\frac{dw}{dt},$$

where dw/dt is the mass change rate per unit time, and *S* is the surface area.

Based on mass loss experiments for Pt and Rh conducted between 1000 °C and 1400 °C, the vapor transport rates for Pt and Rh can be calculated [44]. These data are then fitted to obtain continuous vapor transport rate curves over the temperature range. Since the thermocouple alloy wire exhibits a continuous temperature gradient from the hot end to the cold end, the oxide evaporation rates of Pt and Rh serve as inputs for calculating changes in the Rh mass fraction.

The Rh mass fraction in a unit length of thermocouple alloy wire after vapor transport can be calculated for a given operating temperature *T* and duration Δt. The Rh mass fraction is jointly influenced by the vapor transport of Rh and Pt oxides across different temperatures:

(5) $$c_{\mathrm{degrad}}=\frac{m_{\mathrm{Rh},\Delta t}}{m_{\mathrm{Rh},\Delta t}+m_{\mathrm{Pt},\Delta t}},$$

(6) $$m_{\mathrm{Pt},\Delta t}=m_{\mathrm{Pt},0}-v_{\mathrm{Pt},T}*\Delta t*S,$$

(7) $$m_{\mathrm{Rh},\Delta t}=m_{\mathrm{Rh},0}-v_{\mathrm{Rh},T}*\Delta t*S,$$

where $c_{\mathrm{degrad}}$ is the Rh mass fraction per unit length of alloy wire after evaporation over time Δt; $m_{\mathrm{Pt},\Delta t}$ is the mass of Pt per unit length after Δt; and $m_{\mathrm{Rh},\Delta t}$ is the mass of Rh per unit length after Δt.

### 2.2. Change of Seebeck Coefficient

Pt–Rh thermocouples are standardized into three types: type B, type R, and type S. Based on the composition of the anode and cathode alloy wires, they can be categorized into five distinct wire types: R-type negative (RN, pure Pt), S-type negative (SN, pure Pt), B-type negative (BN, Pt-6%Rh), S-type positive (SP, Pt-10%Rh), R-type positive (RP, Pt-13%Rh), B-type positive (BP, Pt-30%Rh). The Seebeck coefficients for the thermocouple wire pairs BP–Pt, BN–Pt, SP–Pt, and RP–Pt can be obtained from the thermocouple reference table compiled by the National Institute of Standards and Technology [45]. Using the experimentally determined Seebeck coefficient of pure Pt reported by Cusack in [46], the following equation applies:

(8) $$S_{\mathrm{PN}}=S_{\mathrm{Pos}}-S_{\mathrm{Neg}}.$$

**Figure 2:**
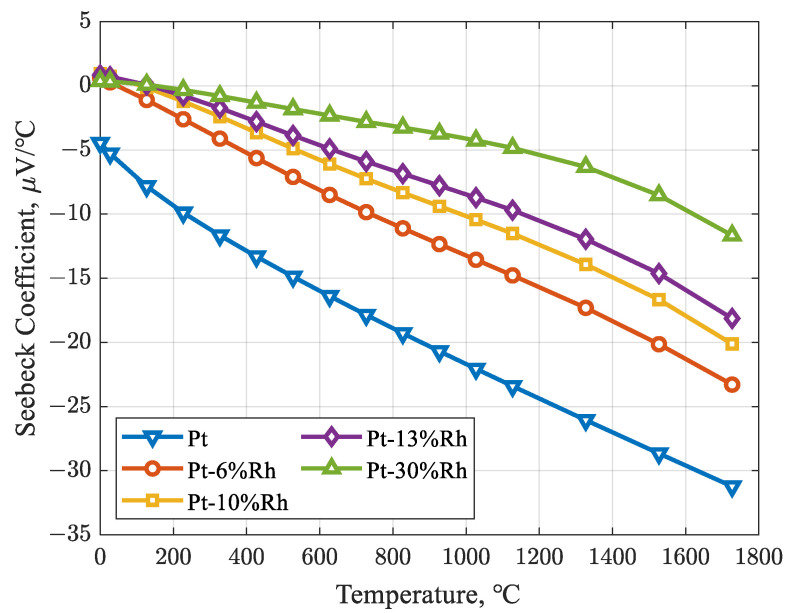
Seebeck coefficient vs. temperature for Pt–Rh thermocouple wires.

The Seebeck coefficients for the BN, SP, RP, and BP alloy wires can thus be determined. Here, $S_{\mathrm{PN}}$ represents the Seebeck coefficient of a thermocouple wire pair, $S_{\mathrm{Pos}}$ denotes the Seebeck coefficient of the anode (positive leg), and $S_{\mathrm{Neg}}$ corresponds to the Seebeck coefficient of the cathode (negative leg). Table 1 presents the Seebeck coefficients of different Pt–Rh thermocouple alloy wires at various temperatures and is illustrated in Figure 2.

**Figure 3:**
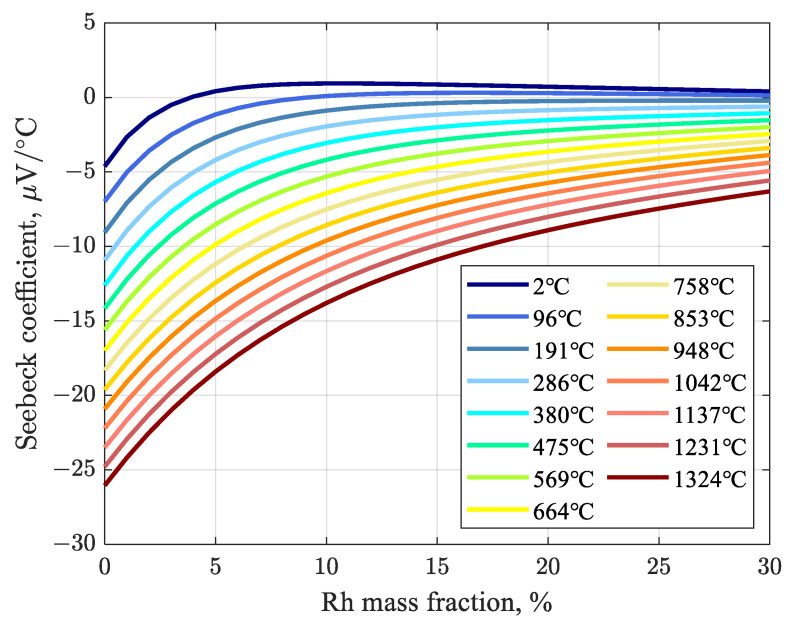
Relationship between Rh mass fraction and Seebeck coefficient at different temperatures.

Through functional fitting, the relationship between the Seebeck coefficient and the Rh mass fraction was established at various temperatures, yielding curves linking temperature, Seebeck coefficient, and Rh mass fraction. The curves are used to compute the Seebeck coefficient as a Pt–Rh thermocouple degrades over time at high temperature due to changes in the Rh mass fraction [47]. The fitted curves relating Rh mass fraction to Seebeck coefficient are shown in Figure 3, where each curve represents the Seebeck coefficient versus Rh mass fraction at a fixed temperature. Such a representation is essential for simulating the continuous-time degradation process of thermocouples. Based on the change in Rh mass fraction caused by vapor transport of metal oxides from the alloy wire, the resulting alteration in thermoelectric homogeneity—and thus in the Seebeck coefficient—can be determined. By updating the Seebeck coefficient for each unit length of the wire, the overall effect of degraded thermoelectric homogeneity can be quantified, enabling the calculation of Emf drift.

According to the Seebeck effect, when two wires of different composition are joined at one end (the hot end) while the free ends are kept at a different temperature (the cold end), an Emf is generated due to the temperature difference. This Emf depends solely on the temperature gradient along the wires,

(9) $$Emf=\int_{T_{\mathrm{cold}}}^{T_{\mathrm{hot}}}\left(S_{\mathrm{Pos}}-S_{\mathrm{Neg}}\right)dT,$$

where $T_{\mathrm{hot}}$, $T_{\mathrm{cold}}$ denote the temperatures at the hot and cold ends, respectively.

As shown in the equation above, the Emf depends only on the temperatures at the two ends of the alloy wires and is independent of their length, diameter, or other geometric properties. Assuming a uniform temperature gradient from the hot end to the cold end, the alloy wire can be divided into unit-length segments to facilitate the calculation of Emf drift due to thermocouple degradation. Since the Emf is determined solely by the temperature difference between the hot and cold junctions, and not by the wire length, the Emf of a thermocouple of any length after degradation can be obtained through discretization of the wire,

(10) $$Emf\approx k\sum_{i=1}^{l-1}\frac{\left(S_{\mathrm{Pos}}\left(l_{i}\right)\right)-S_{\mathrm{Neg}}\left(l_{i}\right)+\left(S_{\mathrm{Pos}}\left(l_{i+1}\right)\right)-S_{\mathrm{Neg}}\left(l_{i+1}\right)}{2},$$

where *k* is the temperature gradient per alloy segment from the hot end to the cold end, in units of °C.

### 2.3. State Space Model

In high-temperature operating environments, Pt–Rh thermocouples undergo very gradual degradation over time. This degradation primarily impacts the Pt–Rh alloy wires, which operate under harsh conditions. In contrast, the secondary instrument—comprising the filtering and amplification circuits—functions in a normal environment and shows no significant degradation. The overall degradation process of a thermocouple instrument can be represented by a state-space model constructed using an empirical nonlinear curve model [48]. Equation (11) presents the curve-fitting model for the output of the Pt–Rh thermocouple after the filtering and amplification stage. The instrument output at time index *k* is denoted as Q(k).

(11) $$Q\left(k\right)=a\left(k\right)e^{b(k)k}+c\left(k\right)e^{d(k)k},$$

where the state space variables of the nonlinear system are as follows:

(12) $$x\left(k\right)={\left[\begin{array}{cccc} a(k) & b(k) & c(k) & d(k) \end{array}\right]}^{T}.$$

The state variables can be expressed as follows:

(13) $$\begin{array}{c} a\left(k+1\right)=a\left(k\right)+w_{a}\left(k\right),w_{a}∼N\left(0,\sigma_{a}\right),\\ b\left(k+1\right)=b\left(k\right)+w_{b}\left(k\right),w_{b}∼N\left(0,\sigma_{b}\right),\\ c\left(k+1\right)=c\left(k\right)+w_{c}\left(k\right),w_{c}∼N\left(0,\sigma_{c}\right),\\ d\left(k+1\right)=d\left(k\right)+w_{d}\left(k\right),w_{d}∼N\left(0,\sigma_{d}\right), \end{array}$$

where N(0,σ) is Gaussian noise with zero mean and standard deviation σ.

Estimating the state variables of the above system yields a function that characterizes the degradation process of the Pt–Rh thermocouple instrument. At the start of prediction, potential future deviations in the thermocouple are computed, enabling the forecasting of RUL.

## 3. Mathematical Analysis

### 3.1. Extended Kalman Filter

KF was originally applicable only to linear dynamic systems. To extend its utility to a wider range of nonlinear systems, nonlinear variants such as EKF were developed, enabling more accurate state tracking, and were widely used for online monitoring. The process can be summarized in two iterative steps: the time update (prediction) and the measurement update (correction). Through repeated cycles of these two steps, real-time state tracking is achieved. By comparing the predicted state for the next time step with the actual measurement and applying feedback-based correction, the error is continuously reduced, allowing the estimated values to converge toward the true state.

KF is known for its simplicity, ease of implementation, and fast computation, making it suitable for real time state estimation. However, its main limitation is its inability to handle nonlinear systems directly. EKF addresses this by applying a linearization technique that approximates the nonlinear filtering problem as a linear one. A nonlinear system can be expressed as follows:

(14) $$\begin{array}{c} \left\{\begin{array}{c} x_{k+1}=f\left(x_{k},u_{k},w_{k}\right)\\ z_{k}=h\left(x_{k},v_{k}\right) \end{array}\right.\\ p(w)∼N(0,Q)\\ p(v)∼N(0,R) \end{array}$$

where $x_{k}$ is the state variable of the nonlinear system; $z_{k}$ is the observation variable; $u_{k}$ is the input variable; *f* is the state nonlinear function; *h* is the measurement nonlinear function; $w_{k}$ and $v_{k}$ are Gaussian white noise with mean zero and covariance *Q* and *R*, respectively.

For the nonlinear system described above, EKF linearizes the nonlinear functions f(∗) and h(∗) using a first-order Taylor expansion, discarding second-order and higher terms. This yields an approximately linear model, which is then processed using the linear KF. However, this linearization approach inevitably introduces errors.

The EKF algorithm consists of the following steps, as shown in Table 2:

Step 1. State one-step estimation:

(15) $${\hat{x}}_{k+1}^{-}=f\left({\hat{x}}_{k},u_{k},0\right),$$

where ${\hat{x}}_{k+1}^{-}$ is the state variable estimation at time k+1.

Step 2. Error covariance prediction:

(16) $$P_{k+1}^{-}=A_{k}P_{k}A_{k}^{T}+W_{k}Q_{k}W_{k}^{T},$$

where $P_{k+1}^{-}$ is the error covariance predicted at time k+1, $A_{k}$ is the Jacobian matrix of the nonlinear function f(∗) evaluated at $\hat{x_{k}}$.

(17) $$A_{k}=\frac{\partial f}{\partial x}{|}_{x={\hat{x}}_{k}}=\left[\begin{array}{cccc} \frac{\partial f_{1}}{\partial x_{1}} & \frac{\partial f_{1}}{\partial x_{2}} & \ldots & \frac{\partial f_{1}}{\partial x_{n}}\\ \frac{\partial f_{2}}{\partial x_{1}} & \frac{\partial f_{2}}{\partial x_{2}} & \ldots & \frac{\partial f_{2}}{\partial x_{n}}\\ \vdots & \vdots & \ddots & \vdots \\ \frac{\partial f_{n}}{\partial x_{1}} & \frac{\partial f_{n}}{\partial x_{2}} & \ldots & \frac{\partial f_{n}}{\partial x_{n}} \end{array}\right]{|}_{x={\hat{x}}_{k}},$$

Step 3. Calculate the Kalman gain:

(18) $$K_{k}=P_{k}^{-}H_{k}^{T}{\left(H_{k}P_{k}^{-}H_{k}^{T}+V_{k}R_{k}V_{k}^{T}\right)}^{-1},$$

where $H_{k}$ is the Jacobian matrix of the nonlinear function h(∗) evaluated at $\hat{x_{k}}$.

(19) $$H_{k}=\frac{\partial h}{\partial x}{|}_{x={\hat{x}}_{k}}=\left[\begin{array}{cccc} \frac{\partial h_{1}}{\partial x_{1}} & \frac{\partial h_{1}}{\partial x_{2}} & \ldots & \frac{\partial h_{1}}{\partial x_{n}}\\ \frac{\partial h_{2}}{\partial x_{1}} & \frac{\partial h_{2}}{\partial x_{2}} & \ldots & \frac{\partial h_{2}}{\partial x_{n}}\\ \vdots & \vdots & \ddots & \vdots \\ \frac{\partial h_{n}}{\partial x_{1}} & \frac{\partial h_{n}}{\partial x_{2}} & \ldots & \frac{\partial h_{n}}{\partial x_{n}} \end{array}\right]{|}_{x={\hat{x}}_{k}},$$

Step 4. Status update:

(20) $${\hat{x}}_{k}={\hat{x}}_{k}^{-}+K_{k}\left(z_{k}-h\left({\hat{x}}_{k}^{-},0\right)\right),$$

Step 5. Covariance update:

(21) $$P_{k}=\left(I-K_{k}H_{k}\right)P_{k}^{-},$$

In the EKF, the process noise covariance *Q* and the measurement noise covariance *R* are typically selected based on the experience of the practitioner. The prior statistical quality of these noise parameters critically influences the accuracy of the estimates. Incorrect noise covariances can degrade estimation precision and may even lead to filter divergence. Since the degradation process of Pt–Rh thermocouples is extremely slow and can be approximated as a linear trend, their output is on the millivolt scale. Consequently, the initial parameters of the double exponential function in Equation (11) have to be set to very small values. Accordingly, when selecting the parameters for *Q* and *R*, care must be taken to avoid introducing excessive noise, which could lead to estimation divergence.

**Table 2:** The EKF algorithm.

Step	Algorithm
1.	Initialize: ${\hat{x}}_{0}$, $P_{0}$, *Q*, *R*
2.	Form the Jacobian matrix
*BEGIN LOOP*	$k=1\overset{+1}{\to }N$
3.	${\hat{x}}_{k+1}^{-}\leftarrow f\left({\hat{x}}_{k},u_{k},0\right)$
4.	$P_{k+1}^{-}\leftarrow A_{k}P_{k}A_{k}^{T}+W_{k}Q_{k}W_{k}^{T}$
5.	$K_{k}\leftarrow P_{k}^{-}H_{k}^{T}{\left(H_{k}P_{k}^{-}H_{k}^{T}+V_{k}R_{k}V_{k}^{T}\right)}^{-1}$
6.	${\hat{x}}_{k}\leftarrow {\hat{x}}_{k}^{-}+K_{k}\left(z_{k}-h\left({\hat{x}}_{k}^{-},0\right)\right)$
7.	$P_{k}\leftarrow \left(I-K_{k}H_{k}\right)P_{k}^{-}$
*END LOOP*	

### 3.2. Back-Propagation Neural Network

Due to the truncation of higher-order terms in the Taylor expansion during linearization, the EKF accumulates errors over time, which can significantly affect prediction accuracy after the prediction phase begins. BPNN is a type of multilayer feedforward neural network trained using the error back-propagation algorithm under supervised learning. Its advantages include: ① Strong nonlinear function approximation capability. By feeding training data into the network, it can construct a nonlinear mapping between input variables and the output. ② Effective learning ability. Through learning from labeled data, it can extract underlying patterns and rules. ③ Good generalization ability. Once trained, the network exhibits strong extrapolation performance when presented with new, unlabeled data. ④ Simple and practical structure. While maintaining high approximation accuracy, BPNN is relatively simple to implement, consisting of an input layer, multiple hidden layers, and an output layer.

Given that thermocouple degradation is an extremely slow process, typically resulting in only minor drift per year, with a largely linear degradation curve and very weak nonlinear characteristics, a four-layer BPNN with two inputs and a single output was designed to suit the practical application scenario. The role of hidden nodes is to enable nonlinear modeling and extract underlying patterns from the input data. If too few hidden nodes are used, the model may underfit and fail to capture meaningful patterns in the data. Conversely, too many hidden nodes can lead to overfitting, causing the model to learn noise rather than generalizable trends. A multiple-layer hidden architecture can capture nonlinear features more effectively than a single-layer hidden architecture. Therefore, the network was configured with three hidden layers, each containing 5, 15, and 10 nodes, respectively. This provides moderate model capacity, sufficient to capture the nonlinear relationships in the input data, while avoiding excessive complexity that could lead to overfitting. This structure allows the neural network to efficiently fit the nonlinear compensation term for the EKF bias while maintaining good generalization performance. The training algorithm employs Bayesian regularization, which introduces a regularization term to penalize large weights, thereby improving generalization. This method also automatically adjusts the regularization parameter to effectively prevent overfitting, making it well-suited for medium-sized datasets.

The learning rate for the BPNN was set to 0.01. Bayesian regularization was employed as the optimizer to prevent overfitting and leverage its strong generalization capability, enabling effective performance even in small-sample learning scenarios. The activation function used is the sigmoid function. Training is conducted for 1000 epochs, and the stopping criterion is set to terminate upon reaching the maximum number of epochs. During BPNN training, the dataset was constructed from the deviation between the actual data, calculated by the Pt–Rh thermocouple degradation model over the first 1000 h, and the EKF estimation curve at the 1000 h mark. The dataset was split into 60% for training, 20% for validation, and 20% for testing.

### 3.3. EKF–BPNN Method

**Figure 4:**
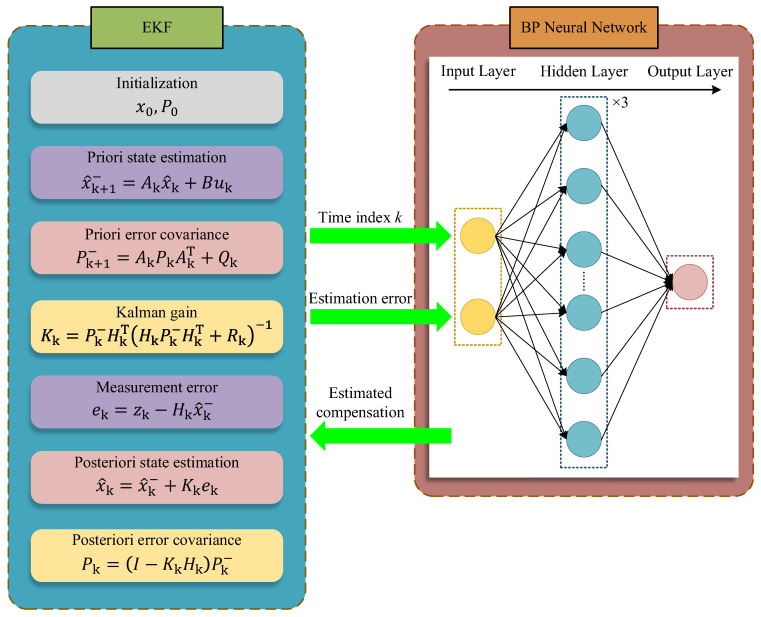
Schema of the proposed degradation estimation method.

During linearization, the EKF neglects higher-order terms in the Taylor expansion of the system state equations, which introduces estimation errors. In the prediction phase, the estimated state obtained through iterative estimation is set into the nonlinear system to forecast the degradation process. Since these errors are not corrected during prediction, they gradually accumulate and significantly distort the predicted trend. This is reflected in the growing deviation between the predicted and actual degradation curves over time. To mitigate the impact of such prediction errors, compensation is applied to the predicted curve. BPNNs have strong capabilities for fitting nonlinear functions. Here, the uncertainties introduced by the linearization of the nonlinear system are approximated using a neural network. The resulting nonlinear mapping between the time index *k* and the required error compensation is then employed to correct errors in the thermocouple degradation prediction. The combined EKF–BPNN approach improves the prediction accuracy of the RUL for Pt–Rh thermocouples. First, the EKF is used to estimate the state of the nonlinear system representing the thermocouple degradation. Next, the latest estimated state is substituted into the state equation to compute the deviation between the estimated and actual degradation values. Finally, the time index *k* and the estimation error are fed into a BPNN to learn the nonlinear mapping for error compensation. Once the prediction of the degradation process begins, the EKF estimated curve is corrected using the BPNN-derived compensation. Figure 4 illustrates the overall framework of the combined algorithm.

The convergence of EKF largely depends on the initial error. Under appropriate conditions, EKF converges locally. Specifically, if the initial error $e\left(0\right)=x\left(0\right)-\hat{x}\left(0\right)$ is sufficiently small, then as t→∞, $e\left(t\right)=x\left(t\right)-\hat{x}\left(t\right)\to 0$ [49]. Regarding the stability of the proposed combined algorithm, the output of the nonlinear mapping learned by BPNN is bounded. Therefore, the global closed-loop stability of the EKF–BPNN algorithm is determined by the global closed-loop stability of the underlying EKF algorithm [50].

## 4. Experimental Analysis and Discussion

### 4.1. Degradation Model Validation of Type B Pt–Rh Thermocouple

Based on the modeling methodology for Pt–Rh thermocouple described in Section 2, a degradation mechanism simulation model was developed for type B Pt–Rh thermocouple, which accounts for the vapor transport rates of Pt and Rh oxides. This model enables the calculation of long-term Emf drift. The validation of the model was conducted in two aspects: ① Verification of the non-degraded state against standard reference values. ② Validation of the degradation model under high temperature operating conditions against actual experimental measurements.

First, consider a type B Pt–Rh thermocouple with a length of 700 mm operating at 1324 °C. Since the Emf of a thermocouple is independent of its length or cross-sectional dimensions and depends only on the temperature at the hot end, the model calculated Emf for the nondegraded state is 8.1308 mV. According to the standard Emf reference table for a cold end temperature of 0 °C published by WIKA Alexander Wiegand SE & Co. KG, Klingenberg, Germany, in Application of Thermocouples (WIKA data sheet IN 00.23, 2016), the standard Emf value at 1324 °C is 8.1115 mV. Comparing the modeled value with the standard reference yields an error of 0.0193 mV, corresponding to a relative error of 0.24%. This verifies the accuracy of the model in simulating the initial, non-degraded state.

**Figure 5:**
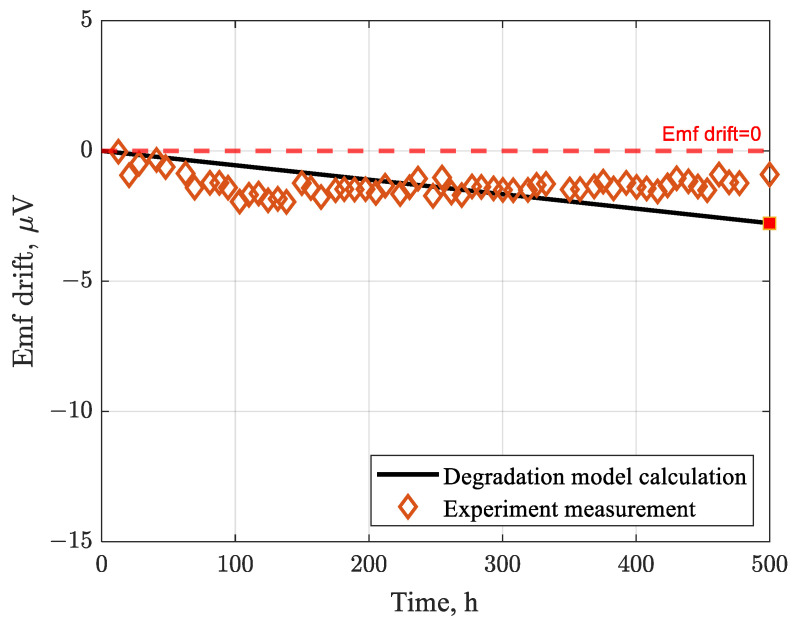
Emf drift of type B Pt–Rh thermocouple after 500 h at 1324 °C.

Next, for a type B Pt–Rh thermocouple with a length of 700 mm, operating continuously at 1324 °C, for 500 h, the degradation model predicts an Emf drift of −2.7752 μV after 500 h (the negative sign indicates a downward drift). This model result is consistent with the experimental measurements reported by Pearce [51], as illustrated in Figure 5. This agreement validates the accuracy of the model in simulating degradation-induced drift.

**Figure 6:**
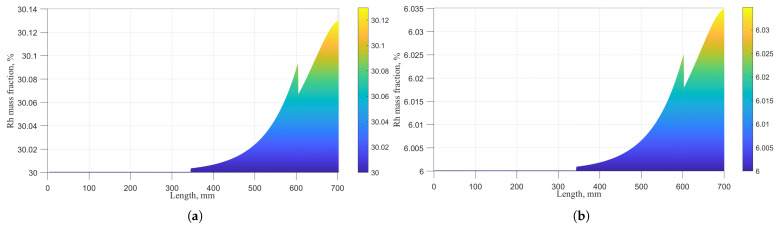
Rh mass fraction of anode and cathode wires after 500 h degradation at 1324 °C: (**a**) Rh mass fraction in Pt-30%Rh wire. (**b**) Rh mass fraction in Pt-6%Rh wire.

As described in Section 2.1, during the degradation of a type B Pt–Rh thermocouple, Pt and Rh in the alloy wires react with oxygen in the air, and the resulting oxides undergo vapor transport at high temperature. This alters the Rh mass fraction in the wires, modifies the Seebeck coefficient, and ultimately leads to Emf drift. However, because the vapor transport temperatures and evaporation rates of Pt and Rh oxides differ, the change in Rh mass fraction is not a continuous process. For a 700 mm type B Pt–Rh thermocouple operating at 1324 °C for 500 h, the variation in Rh mass fraction along the anode (Pt-30%Rh) is shown in Figure 6a, and that along the cathode (Pt-6%Rh) is shown in Figure 6b. The horizontal axis represents the wire length from the hot end to the cold end, where the temperature decreases uniformly. At temperatures below 650 °C, neither Pt nor Rh oxides undergo vapor transport. Accordingly, Figure 6 shows that the Rh mass fraction remains unchanged beyond 343 mm from the hot end. Between 650 °C and 1140 °C, only Pt oxides evaporate, while Rh oxides remain stable. Above 1140 °C, both Pt and Rh oxides participate in vapor transport. As a result, the change in Rh mass fraction at 603 mm is discontinuous.

### 4.2. Pt–Rh Thermocouple Instrument Output

**Figure 7:**
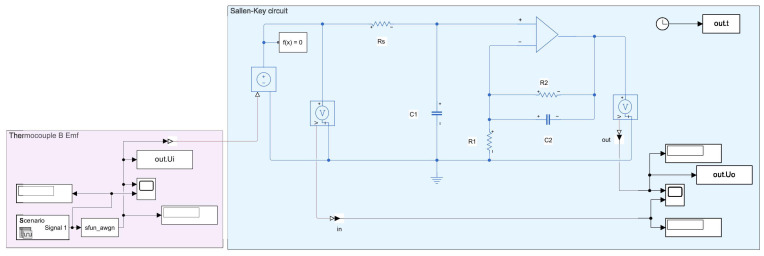
Structure of the Pt–Rh thermocouple filtering and amplification circuit.

Based on the validated Pt–Rh thermocouple degradation model, simulated degradation data over 16,582 h (approximately 1 year, 11 months) were generated using the model. White noise was added to the simulated degradation data, which was then processed by a Sallen–Key circuit for filtering and amplification. After noise removal and amplification, the resulting output represents the analog signal output of the Pt–Rh thermocouple instrument. The procedure is illustrated in Figure 7.

### 4.3. Prediction by EKF–BPNN Compared with Other Algorithms

**Figure 8:**
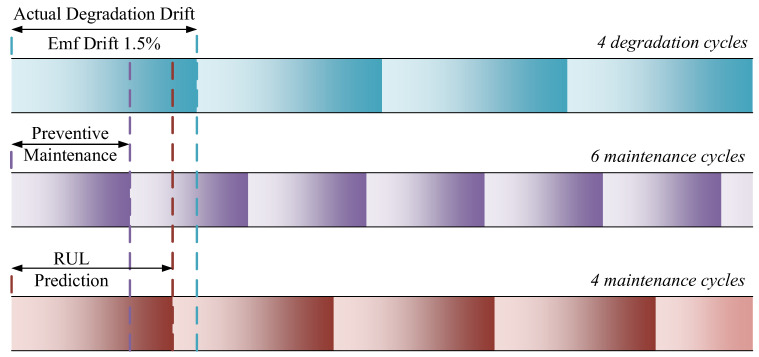
Schematic diagram illustrating the reduction of engineering system operation and maintenance costs through RUL prediction.

In engineering systems with a large number of instruments, a preventive maintenance strategy is typically adopted. This approach involves performing maintenance and calibration well before an instrument reaches its end of life, ensuring its continued reliable operation [52]. For thermocouple instruments, calibration is required once the voltage output drift reaches 1.5%. The practical value of RUL prediction for Pt–Rh thermocouple is illustrated in Figure 8. Over its full lifecycle, a thermocouple may experience actual degradation requiring, for example, four calibration cycles. However, maintenance personnel cannot directly observe the true degradation process and must therefore adopt a conservative preventive maintenance strategy, scheduling calibration earlier than strictly necessary. As a result, the total number of maintenance operations over the instrument’s life may exceed four. By using the RUL prediction method, maintenance can be scheduled based on the predicted degradation degree. This enables the extension of maintenance intervals, thereby reducing high operational and maintenance expenses.

To validate the proposed EKF–BPNN algorithm, comparative simulations were conducted using the EKF, PF, UKF, and EPF algorithms, highlighting the advantages of the EKF–BPNN approach. For a clear performance comparison, identical parameter values were used for the initial state $x_{0}=\left[\begin{array}{cccc} 0.04721 & -6.505e-7 & 3.309e-8 & -0.02738 \end{array}\right]$, process noise covariance $Q_{\mathrm{EKF}/\mathrm{UKF}}=0.3diag\left[\begin{array}{cccc} 10^{-4} & 10^{-6} & 10^{-6} & 10^{-4} \end{array}\right]$, $Q_{\mathrm{PF}/\mathrm{EPF}}=10^{-5}diag\left[\begin{array}{cccc} 10^{-4} & 10^{-8} & 10^{-8} & 10^{-4} \end{array}\right]$, measurement noise covariance $R_{\mathrm{EKF}/\mathrm{UKF}}=10^{-3}$, $R_{\mathrm{PF}/\mathrm{EPF}}=10^{-7}$, initial error covariance $P_{0}=3/4diag\left[\begin{array}{cccc} 1 & 1 & 1 & 1 \end{array}\right]$, as well as the number of particles n=200 across the algorithms.

Based on the degradation model for the type B Pt–Rh thermocouple proposed in Section 2, after operating at 1324 °C for 16,582 h (approximately 1 year 11 months), the output Emf exhibits a negative drift of 1.5%, declining from 8.1308 mV to 8.0090 mV. Following Sallen–Key filtering and amplification, the voltage output of the thermocouple instrument is obtained. Starting the prediction from the 1000 h mark, the actual RUL of the Pt–Rh thermocouple is 15,582 h. The EKF–BPNN prediction algorithm proposed in this paper is compared with the EKF, EPF, UKF, and PF methods. Beginning the prediction at 1000 h and using the 1.5% voltage drift as the calibration threshold, the predicted RUL values obtained are 15,312 h, 14,946 h, 13,136 h, 10,295 h, and 6851 h, respectively. The RUL predicted by EKF–BPNN shows the smallest deviation, which detects the need for maintenance calibration 270 h (approximately 11 days) in advance. In contrast, the other algorithms yield substantially larger deviations from the actual RUL, offering limited practical guidance for maintenance.

**Figure 9:**
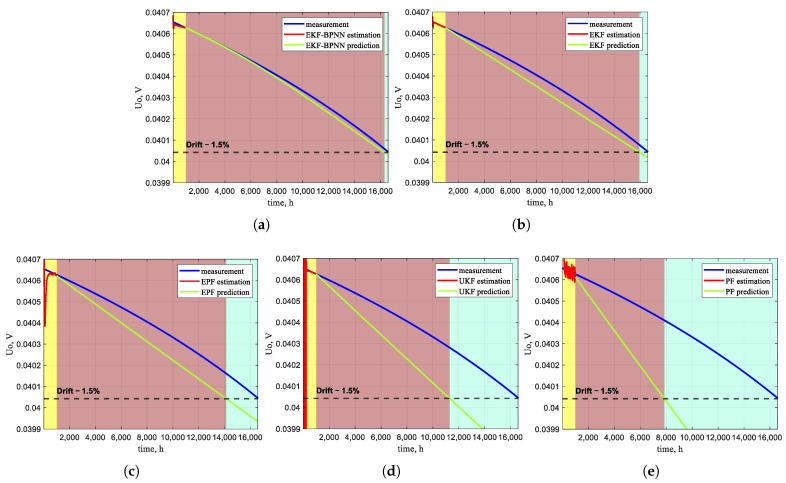
Results for RUL prediction. (**a**) Simulation result of the EKF–BPNN algorithm. (**b**) Simulation result of the EKF algorithm. (**c**) Simulation result of the EPF algorithm. (**d**) Simulation result of the UKF algorithm. (**e**) Simulation result of the PF algorithm.

Figure 9 presents the predictions of EKF–BPNN, EKF, EPF, UKF, and PF for the time at which the Pt–Rh thermocouple reaches a negative drift of 1.5%. The yellow region indicates the state estimation of the degradation model conducted within the first 1000 h; the red region represents the predicted period from 1000 h until the drift reaches 1.5%; and the blue region illustrates the deviation between the predicted and actual RUL. It can be clearly observed that the blue region obtained with the EKF–BPNN algorithm is the smallest, whereas that of the PF algorithm is the largest. This qualitatively demonstrates that EKF–BPNN delivers superior performance in predicting the RUL of the Pt–Rh thermocouple.

**Figure 10:**
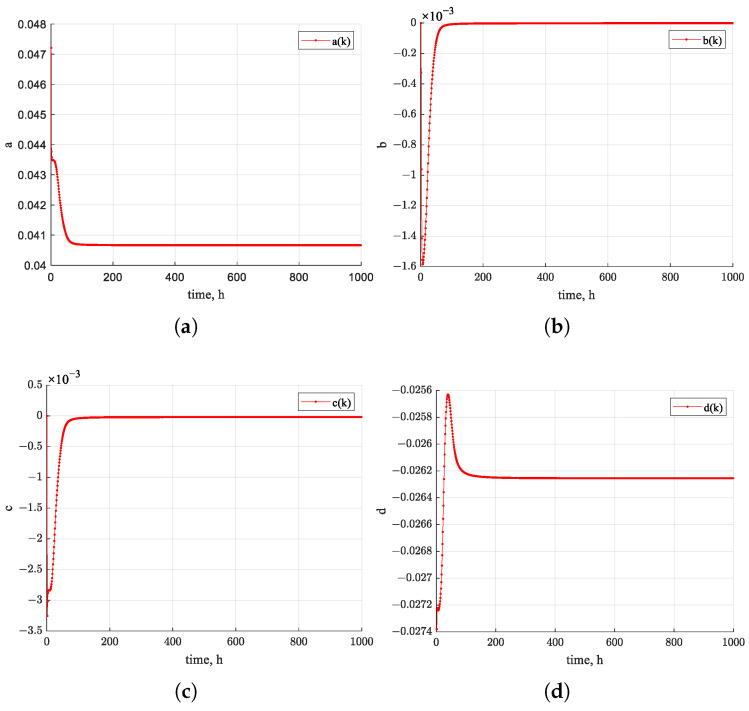
States of EKF algorithm. (**a**) a(k) estimation. (**b**) b(k) estimation. (**c**) c(k) estimation. (**d**) d(k) estimation.

**Figure 11:**
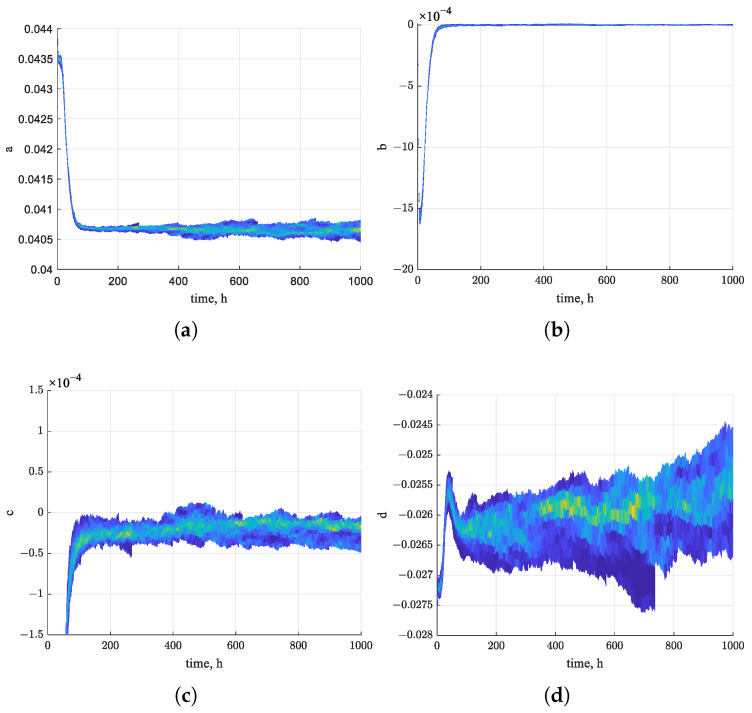
States of the EPF algorithm. (**a**) a(k) estimation of particles. (**b**) b(k) estimation of particles. (**c**) c(k) estimation of particles. (**d**) d(k) estimation of particles.

**Figure 12:**
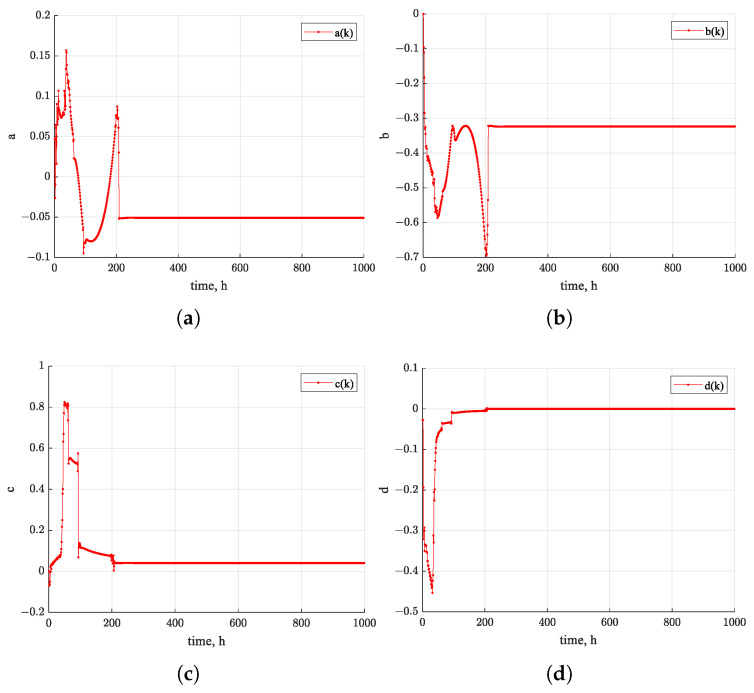
States of the UKF algorithm. (**a**) a(k) estimation. (**b**) b(k) estimation. (**c**) c(k) estimation. (**d**) d(k) estimation.

**Figure 13:**
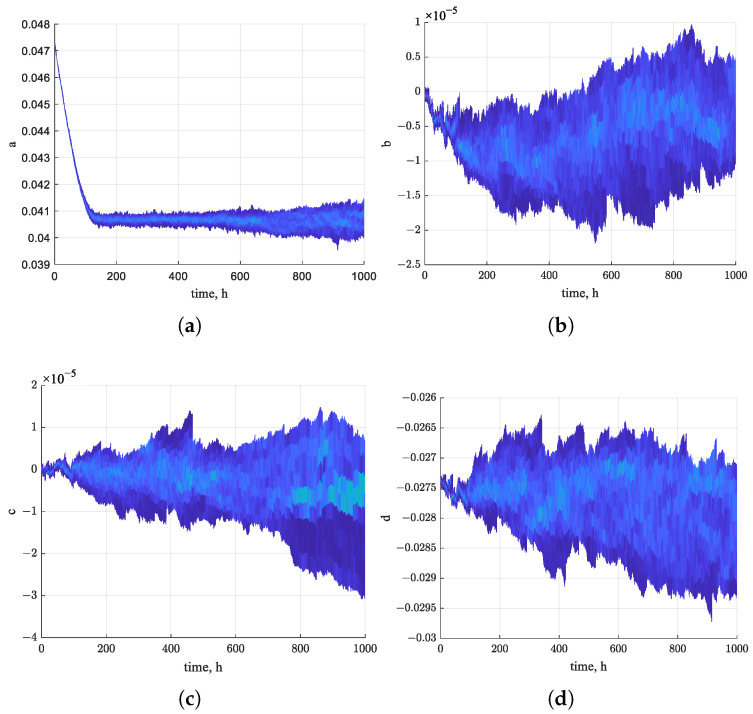
States of the PF algorithm. (**a**) a(k) estimation of particles. (**b**) b(k) estimation of particles. (**c**) c(k) estimation of particles. (**d**) d(k) estimation of particles.

Figure 10 shows the estimated trajectories of the four state variables of the nonlinear system using the EKF algorithm over the first 1000 h. The estimates exhibit a clear trend toward convergence over time. Figure 11, Figure 12 and Figure 13 present the states of EPF, UKF, and PF. In contrast, the estimated state trajectories obtained with the PF algorithms show larger deviations in the distribution of particles used for state estimation. During the estimation process, the particles continuously adjust their distribution to minimize the discrepancy between the nonlinear function value and the actual measurements. In the figures, warmer colors indicate higher particle concentrations, while cooler colors indicate lower concentrations. In comparison, the particle distribution in EPF is more concentrated than that in PF, where particles remain relatively dispersed, and the states fail to converge clearly. As demonstrated in Figure 9, the RUL prediction performance follows the order EKF–BPNN, EKF, EPF, UKF, PF. Therefore, the accuracy of the predicted degradation trend in the Pt–Rh thermocouple instrument output is directly linked to the convergence behavior of the estimated states in the nonlinear system.

For objects like Pt–Rh thermocouples, which degrade very slowly over long time scales, the degradation process exhibits weak nonlinearity over short time intervals and can be approximated as nearly linear. This smoothness arises from the instrument’s two-component structure: the primary instrument and the secondary instrument. The primary instrument, consisting of two different alloy wires, operates directly in harsh environments where external disturbances can introduce high-frequency noise during the conversion of physical variables into electrical signals. However, the secondary instrument incorporates a low-pass filter that effectively removes this high-frequency noise, yielding a clean electrical signal. As a result, the data used for RUL prediction is a smooth curve. Moreover, the degradation of Pt–Rh thermocouples is extremely slow, taking about two years to drift by 1.5%. Over short time intervals, this process can be approximated as linear. This low level of nonlinearity means that the error introduced by the EKF when truncating higher-order terms in the Taylor expansion is small, since the actual curve is smooth and locally linear. In contrast, EPF and PF methods employ hundreds of particles to trace the degradation trend. For such weakly nonlinear systems, the large number of particles can actually introduce additional noise and increased estimation variance, leading to poorer prediction performance than the EKF. UKF employs the unscented transform to propagate the mean and covariance through the nonlinear function. It selects a set of sigma points from the prior state distribution that capture its mean and covariance, then passes these points through the nonlinear function to obtain the transformed mean and covariance. The sigma point selection process can also introduce errors. On the other hand, when the degradation process exhibits strong nonlinearity, EPF and PF methods tend to outperform the EKF and UKF, as their particle-based approach can capture more complex nonlinear patterns. However, EKF introduces significant errors by employing a Taylor expansion and neglecting higher-order terms. Given the specific characteristics of Pt–Rh thermocouples studied here, the EKF provides a reasonably accurate baseline prediction of the degradation trend. Compensating this prediction with the nonlinear error learned by a BPNN further refines the result, yielding a more accurate forecast of Pt–Rh thermocouple degradation.

### 4.4. Results Analysis

To quantitatively compare the performance of each algorithm, four key regression metrics are introduced: MAE, RMSE, MAPE, and $R^{2}$. These metrics are defined as follows [53,54]:

(22) $$MAE=\frac{1}{N}\sum_{i=1}^{N}\left|Emf_{i}-\hat{Emf_{i}}\right|,$$

(23) $$RMSE=\sqrt{\frac{1}{N}\sum_{i=1}^{N}{\left(Emf_{i}-\hat{Emf_{i}}\right)}^{2}},$$

(24) $$MAPE=\frac{1}{N}\sum_{i=1}^{N}\left|\frac{Emf_{i}-\hat{Emf_{i}}}{Emf_{i}}\right|\times 100\%,$$

(25) $$R^{2}=1-\frac{\sum_{i=1}^{N}{\left(Emf_{i}-\hat{Emf_{i}}\right)}^{2}}{\sum_{i=1}^{N}{\left(Emf_{i}-\bar{Emf}\right)}^{2}},$$

where $Emf_{i}$ is the actual Pt–Rh thermocouple Emf, $\hat{Emf_{i}}$ is the predicted Pt–Rh thermocouple Emf, $\bar{Emf}$ is the mean of the actual Pt–Rh thermocouple Emf. For the MAE, RMSE, and MAPE metrics, a smaller value indicates that the algorithm’s output deviates less from the actual Pt–Rh thermocouple Emf, reflecting a better performance. For the $R^{2}$ coefficient, a value closer to 1 signifies better predictive accuracy and thus greater superiority of the algorithm.

To demonstrate that the proposed EKF–BPNN algorithm outperforms the EKF, EPF, UKF, and PF algorithms in predicting the RUL of Pt–Rh thermocouples, and to confirm that this performance advantage is statistically significant rather than the result of random fluctuations or sampling error. Ten repeated trials were conducted, and error metrics were computed. Given the small sample size, a paired *t*-test was applied to evaluate the significance of differences between the error data from the EKF–BPNN and those from the other algorithms. The ten sets of error data used in the *t*-test are shown in Table 3. The following hypotheses are formulated:

$H_{0}$: There is no significant difference between the mean error metrics of the two paired algorithms.

$H_{1}$: There is a significant difference between the mean error metrics of the two paired algorithms.

**Table 3:** The ten sets of error metrics data for the *t*-test.

	MAE/%	RMSE/%	MAPE/%	$R^{2}$
EKF–BPNN1	0.0013	0.0016	0.0326	0.9905
EKF–BPNN2	0.0011	0.0014	0.0283	0.9929
EKF–BPNN3	0.0018	0.0021	0.0446	0.9842
EKF–BPNN4	0.0012	0.0014	0.0290	0.9927
EKF–BPNN5	0.0015	0.0018	0.0376	0.9881
EKF–BPNN6	0.0031	0.0037	0.0763	0.9496
EKF–BPNN7	0.0005	0.0006	0.0118	0.9987
EKF–BPNN8	0.0011	0.0013	0.0273	0.9938
EKF–BPNN9	0.0031	0.0039	0.0781	0.9465
EKF–BPNN10	0.0009	0.0011	0.0229	0.9955
EKF1	0.0042	0.0045	0.1039	0.9277
EKF2	0.0042	0.0045	0.1039	0.9277
EKF3	0.0042	0.0045	0.1039	0.9277
EKF4	0.0042	0.0045	0.1039	0.9277
EKF5	0.0042	0.0045	0.1039	0.9277
EKF6	0.0042	0.0045	0.1039	0.9277
EKF7	0.0042	0.0045	0.1039	0.9277
EKF8	0.0042	0.0045	0.1039	0.9277
EKF9	0.0042	0.0045	0.1039	0.9277
EKF10	0.0042	0.0045	0.1039	0.9277
EPF1	0.0086	0.0092	0.2126	0.6925
EPF2	0.0078	0.0085	0.1940	0.7402
EPF3	0.0078	0.0085	0.1937	0.7412
EPF4	0.0058	0.0062	0.1430	0.8617
EPF5	0.0089	0.0097	0.2217	0.6571
EPF6	0.0091	0.0100	0.2268	0.6404
EPF7	0.0072	0.0078	0.1782	0.7823
EPF8	0.0069	0.0074	0.1702	0.8019
EPF9	0.0091	0.0099	0.2251	0.6464
EPF10	0.0089	0.0097	0.2204	0.6614
UKF1	0.0179	0.0200	0.4439	−0.4388
UKF2	0.0179	0.0200	0.4439	−0.4388
UKF3	0.0179	0.0200	0.4439	−0.4388
UKF4	0.0179	0.0200	0.4439	−0.4388
UKF5	0.0179	0.0200	0.4439	−0.4388
UKF6	0.0179	0.0200	0.4439	−0.4388
UKF7	0.0179	0.0200	0.4439	−0.4388
UKF8	0.0179	0.0200	0.4439	−0.4388
UKF9	0.0179	0.0200	0.4439	−0.4388
UKF10	0.0179	0.0200	0.4439	−0.4388
PF1	0.0397	0.0446	0.9847	−6.1899
PF2	0.0474	0.0532	1.1759	−9.2133
PF3	0.0661	0.0758	1.6423	−19.7456
PF4	0.0389	0.0443	0.9648	−6.0894
PF5	0.0411	0.0468	1.0210	−6.8839
PF6	0.0475	0.0544	1.1803	−9.6856
PF7	0.0387	0.0443	0.9619	−6.0898
PF8	0.0579	0.0665	1.4370	−14.9668
PF9	0.0380	0.0436	0.9441	−5.854
PF10	0.0463	0.0526	1.1489	−8.9838

**Figure 14:**
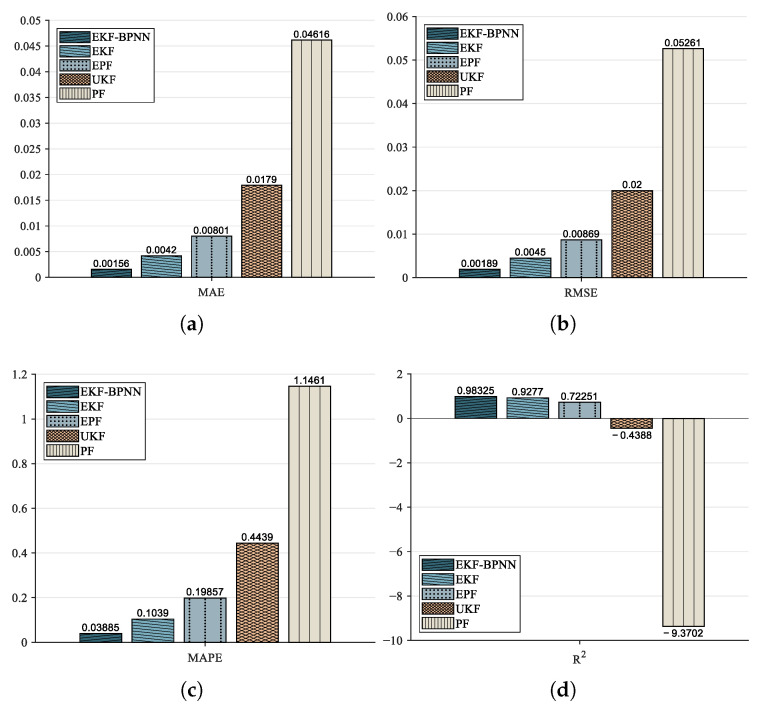
Performance evaluation of different algorithms: (**a**) MAE/%. (**b**) RMSE/%. (**c**) MAPE/%. (**d**) $R^{2}$.

The calculated *p*-values from the *t*-tests are presented in Table 4. Since all *p*-values are below 0.05, the null hypothesis $H_{0}$ is rejected. This indicates a statistically significant difference in the mean error metrics between EKF–BPNN and the other algorithms, confirming that EKF–BPNN achieves superior RUL prediction performance. The averaged MAE, RMSE, MAPE, and $R^{2}$ values across the algorithms are shown in Figure 14. It can be observed that the proposed EKF–BPNN yields lower MAE, RMSE, and MAPE values, while its $R^{2}$ coefficient is the closest to 1.

$R^{2}$ is used to measure how well the algorithm’s predictions fit the observed data. Its theoretical range is [0, 1], with values closer to 1 indicating stronger performance. However, for the RUL prediction results of Pt–Rh thermocouples, the UKF and PF algorithms both yield negative $R^{2}$ values when compared with the actual degradation curve. This indicates that their predictions perform worse than simply using the mean of the target variable, demonstrating that these algorithms are highly unsuitable for the RUL prediction problem of Pt–Rh thermocouples, as their outputs deviate further from the true values than the mean does. Therefore, the UKF and PF algorithms are not appropriate for the RUL prediction of Pt–Rh thermocouple degradation.

## 5. Conclusions

To address the long-term performance degradation and RUL prediction of Pt–Rh thermocouples in harsh industrial environments, and given the extremely slow drift characteristics of Emf, this paper proposes a degradation trend prediction algorithm that combines the EKF with a BPNN. First, based on the Seebeck effect and vapor transport theory, a degradation model for type B Pt–Rh thermocouples was established and validated against experimental data. Second, to mitigate the errors introduced by the linearization step in the EKF, an EKF algorithm compensated by a BPNN was developed. This hybrid approach leverages the strengths of both filtering algorithms and neural networks to achieve high accuracy in RUL prediction. Finally, a comparative analysis with other algorithms was conducted to comprehensively evaluate the RUL prediction performance of the proposed EKF–BPNN method.

The experimental results demonstrate that the proposed algorithm achieves high prediction accuracy for instruments that exhibit slow, long-term degradation drift, such as Pt–Rh thermocouples. For weakly nonlinear systems that can be approximated as linear over short time scales, algorithms with strong nonlinear feature extraction capabilities do not perform ideally. Although linearizing the nonlinear system introduces errors, the BPNN-based compensation effectively corrects these errors, leading to improved prediction accuracy of the degradation trend.

Building upon the type B Pt–Rh thermocouple degradation model proposed at 1324 °C, future research should focus on experimentally obtaining long-term degradation data over a wider temperature range. These data will be used to calibrate the current model, thereby enhancing its adaptability across a broader range of thermal conditions. Furthermore, subsequent research will further explore the differences between data-driven and model-based RUL prediction methods in terms of applicability to different systems and performance.

## Figures and Tables

**Table 1:** Seebeck coefficient of Pt–Rh thermocouple alloy wires.

Temperature (°C)	Pt	Pt-6%Rh	Pt-10%Rh	Pt-13%Rh	Pt-30%Rh
−173	4.29	-	-	-	-
−123	1.32	-	-	-	-
−73	−1.27	-	-	-	-
0	−4.45	0.619	0.953	0.84	0.373
27	−5.28	0.272	0.755	0.712	0.342
127	−7.83	−1.1	−0.128	0.08	0.099
227	−9.89	−2.602	−1.215	−0.769	−0.32
327	−11.66	−4.117	−2.394	−1.731	−0.791
427	−13.31	−5.633	−3.646	−2.79	−1.303
527	−14.88	−7.101	−4.896	−3.866	−1.812
627	−16.39	−8.503	−6.098	−4.906	−2.307
727	−17.86	−9.847	−7.242	−5.9	−2.808
827	−19.29	−11.108	−8.327	−6.848	−3.251
927	−20.69	−12.34	−9.386	−7.781	−3.728
1027	−22.06	−13.557	−10.436	−8.715	−4.253
1127	−23.41	−14.776	−11.513	−9.69	−4.841
1327	−26.06	−17.306	−13.924	−11.956	−6.319
1527	−28.66	−20.141	−16.664	−14.634	−8.53
1727	−31.23	−23.292	−20.12	−18.134	−11.658

**Table 4:** The *p*-value of the *t*-test.

	EKF–BPNN vs. EKF	EKF–BPNN vs. EPF	EKF–BPNN vs. UKF	EKF–BPNN vs. PF
MAE	5.59e−6	6.26e−9	6.13e−13	1.07e−7
RMSE	3.27e−5	1.11e−8	1.56e−12	1.30e−7
MAPE	6.17e−6	6.10e−9	6.48e−13	1.08e−7
$R^{2}$	6.79e−6	5.65e−7	2.16e−18	5.46e−5

## Data Availability

In this paper, the research data are shared via the open database Zendo through the following DOI: https://doi.org/10.5281/zenodo.18278065.

## Abbreviations

The following abbreviations are used in this manuscript:

BPNN	Back-propagation neural network
EKF	Extended Kalman filter
Emf	Electromotive force
EPF	Extended Kalman particle filter
KF	Kalman filter
PF	Particle filter
Pt	Platinum
Rh	Rhodium
RUL	Remaining useful life
UKF	Unscented Kalman filter
